# Classification and treatment of proximal humerus fractures: inter-observer reliability and agreement across imaging modalities and experience

**DOI:** 10.1186/1749-799X-6-38

**Published:** 2011-07-29

**Authors:** Abtin Foroohar, Rick Tosti, John M Richmond, John P Gaughan, Asif M Ilyas

**Affiliations:** 1 Department of Orthopaedic Surgery and Sports Medicine, Temple University School of Medicine, 3401 N. Broad Street, Philadelphia, PA 1914, USA; 2 Department Of Physiology, Temple University School of Medicine, 3500 N. Broad Street, Philadelphia, PA 19141, USA; 3 Rothman Institute, Thomas Jefferson University Hospital, 925 Chestnut Street, Philadelphia, PA 19107, USA

## Abstract

**Summary:**

Proximal humerus fractures (PHF) are common injuries, but previous studies have documented poor inter-observer reliability in fracture classification. This disparity has been attributed to multiple variables including poor imaging studies and inadequate surgeon experience. The purpose of this study is to evaluate whether inter-observer agreement can be improved with the application of multiple imaging modalities including X-ray, CT, and 3D CT reconstructions, stratified by physician experience, for both classification and treatment of PHFs.

**Methods:**

Inter-observer agreement was measured for classification and treatment of PHFs. A total of sixteen fractures were imaged by plain X-ray (scapular AP and lateral), CT scan, and 3D CT reconstruction, yielding 48 randomized image sets. The observers consisted of 16 orthopaedic surgeons (4 upper extremity specialists, 4 general orthopedists, 4 senior residents, 4 junior residents), who were asked to classify each image set using the Neer system, and recommend treatment from four pre-selected choices. The results were evaluated by kappa reliability coefficients for inter-observer agreement between all imaging modalities and sub-divided by: fracture type and observer experience.

**Results:**

All kappa values ranged from "slight" to "moderate" (k = .03 to .57) agreement. For overall classification and treatment, no advanced imaging modality had significantly higher scores than X-ray. However, when sub-divided by experience, 3D reconstruction and CT scan both had significantly higher agreement on classification than X-ray, among upper extremity specialists. Agreement on treatment among upper extremity specialists was best with CT scan. No other experience sub-division had significantly different kappa scores. When sub-divided by fracture type, CT scan and 3D reconstruction had higher scores than X-ray for classification only in 4-part fractures. Agreement on treatment of 4 part fractures was best with CT scan. No other fracture type sub-division had significantly different kappa scores.

**Conclusions:**

Although 3D reconstruction showed a slight improvement in the inter-observer agreement for fracture classification among specialized upper extremity surgeons compared to all imaging modalities, fracture types, and surgeon experience; overall all imaging modalities continue to yield low inter-observer agreement for both classification and treatment regardless of physician experience.

## Introduction

Proximal humeral fractures (PHFs) comprise 5% of all fractures in adults and are the third most common fracture in adults over 65 years old [1]. In 1970, Charles Neer II created a classification system for fractures of the proximal humerus, which is widely utilized [2,3]. However, over the past 2 decades the reliability of Neer's system has been challenged, as multiple studies have reported low inter-observer agreement when attempting to classify PHFs using Neer's system [4-17] or recommending subsequent treatment [18]. Neer's classification is not alone in this quandary, as many studies have similarly reported disagreement in classification schemes for other types of fractures [19-21]. It has been postulated that the low levels of agreement is not a limitation of the classification systems itself but rather the surgeons' inability to accurately interpret the images. In fact, Neer himself has rebutted that experience and suboptimal imaging are likely responsible for the lack of agreement in his system [22].

Although some authors have evaluated the effect on inter-observer agreement by adding advanced imaging such as CT scans and three-dimensional (3D) reconstructions, [4,10,14,15,23-25] the results have been inconclusive, and none have addressed all of these modalities in terms of both classification and treatment recommendations as a function of physician experience. Thus, to the best of our knowledge, this is the first study to evaluate the inter-observer agreement of multiple imaging modalities: X-ray, CT, and 3D reconstructions on both the classification as well as treatment of proximal humerus fractures in a single study. The secondary study goal was to observe the effect of stratifying agreement based on fracture severity and surgeon experience.

## Patients and methods

Sixteen proximal humerus fractures were selected and classified by the senior author as four 2-part fractures, eight 3-part fractures, and four 4-part fractures. Each of the 16 fractures had an X-ray (anteroposterior and a scapular-Y lateral), a CT scan, and a 3D CT reconstruction, which resulted in a total of 48 standardized image sets. All images were taken between 2003-2008 at the same institution and drawn from the same PACS system.

After providing a brief review of the Neer classification system, each observer was presented the same 48 image-sets by PowerPoint in random order. They were blinded to any patient demographic information, mechanism of injury, or associated morbidities. Each observer was asked only two questions per set of images: (1) to classify the fracture using the Neer classification, and (2) to determine their treatment of choice. Treatment options were standardized to four choices: non-operative, open reduction internal fixation, hemiarthroplasty or total shoulder arthroplasty. No case demographics were provided.

The observers included orthopedists of varying experience: 8 board-certified attending surgeons (consisting of 4 general orthopedists and 4 upper extremity specialists), 4 senior residents, and 4 junior residents. A general orthopedist was defined as a surgeon practicing all aspects of orthopaedic surgery including the surgical management of PHFs. An upper extremity specialist was defined as a surgeon with fellowship training and a practice focus on the upper extremity whose practice includes the surgical management of PHFs.

### Statistical Analysis

Inter-observer agreement was assessed via computer-calculated kappa statistics based on the works of Cohen and Fleiss [26,27]. Calculating agreement by this method adjusts the proportion of observed agreement between observers to correct for the proportion of agreement between observers due to chance. Thus, kappa values are always lower than absolute agreement except when 100% agreement is achieved. The kappa coefficients range from +1 (total agreement) to 0 (chance agreement). Although kappa values ranging 0 to -1 are possible, these seldom are encountered, as it represents an agreement less than that which would occur by random chance. The strength of agreement of kappa coefficients was guided by the boundaries suggested by Landis and Koch [28]. Values less than 0.00 indicate "poor" reliability, 0.00-0.20 is "slight" reliability, 0.21-0.40 is "fair" reliability, 0.41-0.60 is "moderate" reliability, 0.61-0.80 is "substantial" agreement, 0.81-1.00 "excellent" or "almost perfect" agreement. Although these categories are arbitrary, they have been well recognized in the orthopedic literature. Statistical differences between individual kappa values were considered significant when the upper and lower boundaries of 95% confidence intervals did not overlap.

## Results

### Overall inter-observer agreement (table 1, figure 1)

**Table 1:** Overall inter-observer agreement

	Classification	Classification		Treatment		
**Modality**	**Kappa Score**	**95% Confidence Interval**	**Strength of Agreement**	**Kappa Score**	**95% Confidence Interval**	**Strength of Agreement**

Plain film X-ray	0.1416	(0.1177-0.1655)	slight	0.2852	(0.2600-0.3104)	fair
2D CT scan	0.0690	(0.0000-0.0920)	slight	0.3285	(0.3028-0.3543)	fair
3D reconstruction	0.0947	(0.0710-0.1185)	slight	0.3082	(0.2817-0.3346)	fair

**Figure 1:**
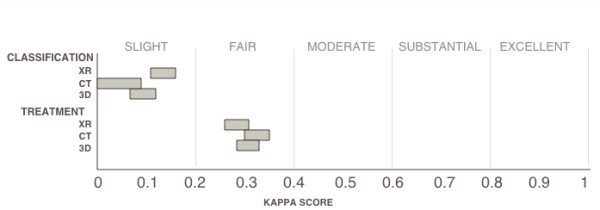
**Graph showing confidence intervals of overall kappa scores for classification and treatment of proximal humerus fractures**. Inter-observer agreement was considered significantly different in non-overlapping intervals. Strength of agreement based on guidelines recommended by Landis and Koch [19].

Agreement of classification across all modalities was only "slight," and agreement of treatment across all modalities was "fair." For classification: X-ray > 3D CT reconstruction > 2D CT scan with the kappa values being 0.14, 0.09, 0.07 respectively; 3D reconstruction was not statistically different than either X-ray or CT scan, but X-ray was significantly stronger than CT. For treatment recommendation, the inter-observer agreement ranged from 0.29-0.33, and no statistically significant difference was detected between the modalities.

### Inter-observer agreement subdivided by fracture type (table 2, figure 2)

**Table 2:** Inter-observer agreement subdivided by fracture type

2 Part Neer Fractures						
	**Classification**		**Treatment**			

**Modality**	**Kappa Score**	**95% Confidence Interval**	**Strength of Agreement**	**Kappa Score**	**95% Confidence Interval**	**Strength of Agreement**

Plain film X-ray	0.0358	(0.0000-0.0907)	slight	0.1494	(0.0959-0.2030)	slight
2D CT scan	0.0448	(0.0000-0.0977)	slight	0.2446	(0.1912-0.2980)	fair
3D reconstruction	0.0770	(0.0251-0.1290)	slight	0.1793	(0.1241-0.2344)	slight
						
**3 Part Neer Fractures**						
	**Classification**		**Treatment**			
**Modality**	**Kappa Score**	**95% Confidence Interval**	**Strength of Agreement**	**Kappa Score**	**95% Confidence Interval**	**Strength of Agreement**
Plain film X-ray	0.0877	(0.0556-0.1198)	slight	0.3430	(0.3057-0.3802)	fair
2D CT scan	0.0524	(0.0000-0.0850)	slight	0.2860	(0.2492-0.3229)	fair
3D reconstruction	0.0960	(0.0640-0.1284)	slight	0.3579	(0.3222-0.3935)	fair
						
**4 Part Neer Fractures**						
	**Classification**		**Treatment**			
**Modality**	**Kappa Score**	**95% Confidence Interval**	**Strength of Agreement**	**Kappa Score**	**95% Confidence Interval**	**Strength of Agreement**
Plain film X-ray	0.2600	(0.2105-0.3090)	fair	0.1697	(0.1481-0.2454)	slight
2D CT scan	0.4467	(0.3989-0.4946)	moderate	0.3368	(0.2857-0.3880)	fair
3D reconstruction	0.5743	(0.5225-0.6260)	moderate	0.0893	(0.0344-0.1441)	slight

**Figure 2:**
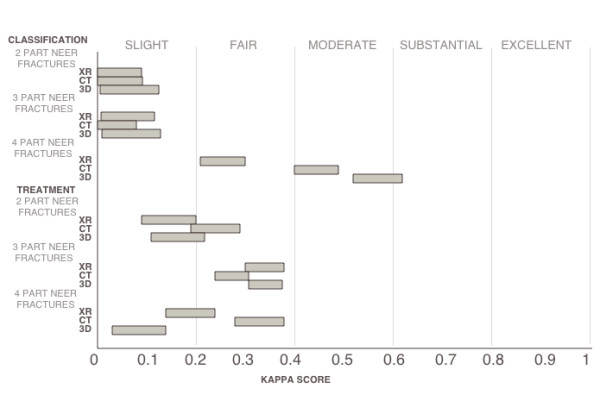
**Graph showing confidence intervals of kappa scores sub-divided by fracture type**. Advanced imaging seemed to only improve agreement of classification in 4 part fractures.

We selected four fractures for each of the four major types of Neer classification schemes yielding a total of sixteen fractures. For classification of 2 part fractures, the kappa values ranged from 0.03-0.07 (achieving "slight" agreement) with no statistically significant differences between the modalities. For treatment of 2 part fractures, the kappa values ranged from 0.15-.24; CT scan was the only modality to reach "fair" agreement, but none of the agreement scores were statistically different from each other. For classification of 3 part fractures, all of the modalities reached only "slight" agreement, and none were statistically different from one another. For treatment of 3 part fractures, all of the modalities reached "fair" agreement, and none were statistically different from one another. For classification of 4 part fractures: 3D reconstruction > CT scan > X-ray, and both 3D reconstruction and CT scan reached the "moderate" level. All kappa values from the 4-part classification subdivision were significantly different. Noteworthy, the highest individual kappa value achieved in this study was agreement on 3D reconstructed 4 part fractures. For treatment of 4 part fractures, CT scan had the highest agreement with a "fair" score of 0.34. This kappa score was statistically different than both of the "slight" scores yielded by X-ray and 3D reconstruction.

### Inter-observer agreement subdivided by experience (table 3, figure 3)

**Table 3:** Inter-observer agreement subdivided by experience

Upper Extremity Specialists						
	**Classification**			**Treatment**		

**Modality**	**Kappa Score**	**95% Confidence Interval**	**Strength of Agreement**	**Kappa Score**	**95% Confidence Interval**	**Strength of Agreement**

Plain film X-ray	0.0315	(0.0000-0.0917)	slight	0.1605	(0.0190-0.3020)	slight
2D CT scan	0.233	(0.1096-0.3339)	fair	0.4673	(0.3294-0.6052)	moderate
3D reconstruction	0.3246	(0.1946-0.4546)	fair	0.1832	(0.0333-0.3330)	slight
						
**General Orthopedists**						
	**Classification**			**Treatment**		
**Modality**	**Kappa Score**	**95% Confidence Interval**	**Strength of Agreement**	**Kappa Score**	**95% Confidence Interval**	**Strength of Agreement**
Plain film X-ray	0.1079	(0.0467-.01691)	slight	0.3883	(0.3213-0.4553)	fair
2D CT scan	0.0351	(0.0000-0.0914)	slight	0.46	(0.3945-0.5255)	moderate
3D reconstruction	0.036	(0.0000-0.0980)	slight	0.4069	(0.3405-0.4734)	moderate
						
**Senior Residents**						
	**Classification**			**Treatment**		
**Modality**	**Kappa Score**	**95% Confidence Interval**	**Strength of Agreement**	**Kappa Score**	**95% Confidence Interval**	**Strength of Agreement**
Plain film X-ray	0.2184	(0.0760-0.3608)	fair	0.4273	(0.2849-0.5697)	moderate
2D CT scan	0.0597	(0.0000-0.2230)	slight	0.2613	(0.1133-0.4094)	fair
3D reconstruction	0.0364	(0.0000-0.1670)	slight	0.368	(0.2154-0.5210)	fair
						
**Junior Residents**						
	**Classification**			**Treatment**		
**Modality**	**Kappa Score**	**95% Confidence Interval**	**Strength of Agreement**	**Kappa Score**	**95% Confidence Interval**	**Strength of Agreement**
Plain film X-ray	0.0295	(0.0000-0.1807)	slight	0.029	(0.0000-0.1701)	slight
2D CT scan	0.1111	(0.0388-0.2610)	slight	0.1288	(0.0397-0.2973)	slight
3D reconstruction	0.1438	(0.0390-0.2915)	slight	0.2284	(0.0738-0.3831)	fair

**Figure 3:**
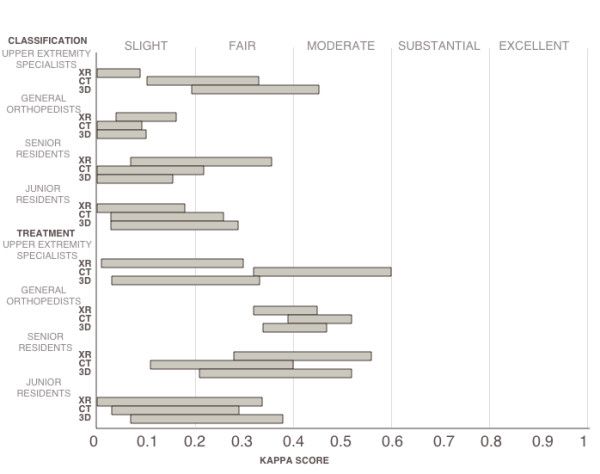
**Graph showing confidence intervals of kappa scores sub-divided by surgeon experience**. Statistically significant differences were only observed among upper extremity surgeons.

We divided our orthopedic observers into upper extremity specialists, general orthopedists, senior residents, and junior residents. Among the upper extremity surgeons, both 3D reconstruction and CT scan yielded "fair" agreement for classification and were both significantly stronger than X-ray. Their agreement trended: 3D reconstruction > CT scan > X-ray. However, CT scan had the highest kappa score for treatment recommendation with a "moderate" score of 0.47. Although CT scan was significantly higher than X-ray in the treatment category, it was not significantly higher than 3D reconstruction. Among general orthopedists, all modalities achieved a "slight" agreement rating (0.04-0.11) for classification, and they ranged from "fair" to "moderate" (0.39-0.46) for the treatment recommendations. No statistically significant differences between any kappa values were observed for the general orthopedists within respective classification or treatment categories. Among senior residents, the kappa scores ranged from "slight" to "fare" (0.03-0.21) for classification and from "fair" to "moderate" (0.26-0.43) for treatment recommendation. No statistically significant differences between any kappa values were observed for the senior residents within respective classification or treatment categories. Among junior residents, all imaging modalities yielded only "slight agreement" for both classification and treatment except one "fair" agreement was observed for treatment recommendations after 3D reconstruction. No statistically significant differences were detected between any kappa scores for the junior residents.

## Discussion

In the past two decades, the validity and reproducibility of fracture classification systems has come under greater scrutiny, which has sparked much debate in the orthopedic literature [29-34]. As a result, subsequent studies have examined the utility of advanced imaging techniques in improving inter-observer agreement, but the results have been inconclusive [4,10,14,15,22,25]. In the beginning of this debate, a few studies have concluded that CT scan or three-dimensional reconstruction add very little in pre-operative assessment [4,10,14,15,22]; however, a study published recently by Brunner et al. has challenged this assertion by showing a consistent increase in inter-observer agreement through the use of stereo-visualization and real 3D imaging [25].

In our series, we examined the effect of advanced imaging, including 3D reconstructions, on fracture classification and treatment and found that inter-observer agreement was less than ideal for both classification and treatment among orthopedic surgeons, which is consistent with the majority of reports in the literature [4-20,22]. In a recent review, "slight" to "moderate" agreement has been reported in almost all major studies regarding inter-observer reliability in PHFs [8], and our results indicate the same despite the addition of advanced imaging in the form of 3D reconstructions. However, it should be noted that the comparison of kappa coefficients across studies should be done with caution, as factors such as bias, prevalence, and marginal distributions influence kappa values and can vary at different institutions [35]. Thus, our second goal was to compare overall inter-observer agreement only within our institution and to observe the effect sub-dividing our results by fracture type and observer experience. In doing this, we observed two major trends in our data: 1) the only significant improvement in agreement with advanced imaging was among upper extremity surgeons and 2) the only benefit of advanced imaging was among all users in attempting to classify 4 part fractures. No benefit was witnessed with advanced imaging in order to improve inter-observer agreement on treatment.

**Figure 4:**
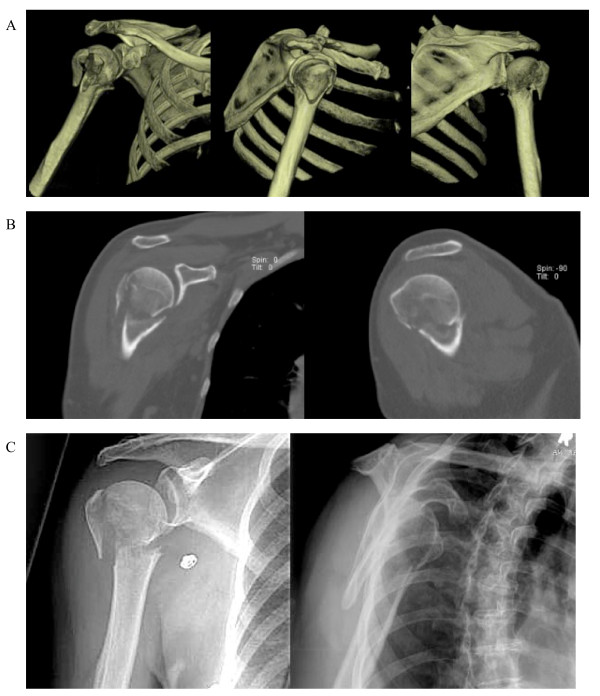
**An example of a (A) 3D reconstruction showing AP, lateral, and PA views of a right shoulder**. Most popular answers: 50% of raters classified this image as a 3-part fracture (37.5% classified 4-part) and 75% recommended ORIF. From the same patient are (B) coronal and axial views of the CT scan with (C) AP and lateral X-rays.

Our study showed that the greatest inter-observer agreement was among upper extremity surgeons with 3D reconstruction. Furthermore, none of the other groups of observers had significantly improved kappa scores with the addition of advanced imaging, which may suggest that experience enhances inter-observer agreement in our study. Reports in the literature are nearly split regarding the role of experience. Kristiansen et al. was the first to suggest that low experience accounted for low agreement [17]. Then, Sidor et al. argued against experience by concluding that the three attending physicians had the same agreement as the residents; however, the group of attending physicians was heterogeneous and not all were orthopedic surgeons [11]. Siebenrock et al. studied only shoulder specialists and found that inter-observer agreement with plain films still landed in the "fair" to "moderate" range; they suggested that experience did not improve the kappa score when compared to other studies, but they did not compare the specialists to a control group [13]. Sallay et al. was the first article to refute experience by sorting observers into groups. They measured agreement with both X-ray and 3D reconstructions, but their technology for 3D reconstruction was an earlier version and had lower resolution than in the present study (Figure 4) [10]. On the other hand, a few studies have supported the role of experience. Brorson et al. showed significantly higher confidence intervals in specialists when compared to residents and fellows, and although not explicitly stated as a study aim, Bernstein et al. showed higher absolute kappa values among attending surgeons when compared to residents [4,7,8]. As a response to the challenge of the 4-type classification system, Neer commented that inter-observer variability is likely the combination of "suboptimal quality of current imaging and inexperienced interpreters [22]." Our study agrees with Neer's interpretation, as our highest and most significant agreement was observed in both our most experienced observers and most advanced imaging modality. This may suggest that the greatest benefit of advanced imaging is to the upper extremity surgeon; however, despite the improved trend, overall agreement is still less than ideal and therefore not recommended.

4-part fractures showed the greatest inter-observer agreement among all observers for both classification and treatment. The data in this category also trended significantly, as 3D reconstruction was stronger than CT scan, which was stronger than X-ray. However, treatment of 4 part-fractures was most agreeable with CT scan. All other subdivisions of fracture type did not show significant improvement with advanced imaging. These data may suggest that complex 4-part fracture classification could be improved by 3D reconstruction. A few studies have corroborated this assertion: Mora-Guix et al. showed that despite the little overall value of CT imaging, it did improve identification of number of fragments [23]. Additionally, Brien et al. cited that the largest point of contention in their inter-observer study was agreeing upon 4-part fractures, and the surgeons would benefit from CT scans in that regard [5].

The literature regarding inter-observer agreement of fracture classifications appears to converge on the following paradigm: low inter-observer agreement is largely caused by compromised interpretation of the imaging, which is caused by imprecise measurements of the pathoanatomy. Moreover, Neer described patient, procedural, and clinical variability as causes of these imprecise measurements [22], and a few studies have improved precision through education [6,8,16]. Important to remember is that "the 4-segment classification is not a radiographic system but is a pathoanatomic classification of fracture displacement [22]." Our study and others have underscored the difficulty in categorizing a 3D concept with 3D images displayed on a 2D screen. Perhaps further studies with experienced users of advanced technology or stereo-visualization need to be evaluated for observer agreement possibly with correlation to intra-operative findings.

There were several study limitations. First, it should be understood that these conclusions are based on an experimental model; thus the distribution of Neer Fractures is not reflective of that which would be experienced in a clinical setting. Furthermore, the observers were not privileged to any patient demographic information, which certainly influences a treating surgeon's decision algorithm. Further studies evaluating agreement of treatment based on a more complete clinical picture would have a broader application. The number of cases (sixteen) presented to the observers was a limitation, and a power analysis was not performed in the selection of this number; however, it was chosen to provide an adequate breadth of cases without resulting in observer fatigue, which might have confounded the results. Additionally, the observers were not able to combine or manipulate images, as they might in a clinical setting. Gonimeters or rulers were also not provided but have been shown to be ignored in clinical setting even when available [11]. The image sets were pre-selected, which imparts a selection bias. Also, treatment comparisons are inherently biased by the observer's comfort level with a procedure and by their experience with the fracture classification, which also may have changed if they were given the opportunity to combine modalities. Observers may have also been limited by their specific experience with 3D technology.

## Summary

In examining the inter-observer agreement with kappa values for X-ray, CT scan, and 3D reconstruction for fracture classification and treatment, we conclude that although 3D reconstruction showed a slight improvement in the inter-observer agreement for fracture classification among specialized upper extremity surgeons, overall all imaging modalities yielded low inter-observer agreement for both classification and treatment.

## Competing interests

The authors declare that they have no competing interests.

## Authors' contributions

AF conceived the study design and participated in data collection. RT wrote the manuscript, constructed the tables and graphs, edited the imaging, revised the statistical methods, and performed the literature search. JR participated in data collection. JG performed the statistical analysis. AI revised the final manuscript, revised the study design, and oversaw all aspects pertaining to the current study. All authors read and approved the final manuscript.
